# Prediction of Technical State of Mechanical Systems Based on Interpretive Neural Network Model

**DOI:** 10.3390/s23041892

**Published:** 2023-02-08

**Authors:** Evgeniy Kononov, Andrey Klyuev, Mikhail Tashkinov

**Affiliations:** 1 Laboratory of Mechanics of Biocompatible Materials and Devices, Perm National Research Polytechnic University, 29 Komsomolsky Prospekt, 614990 Perm, Russia; 2 Faculty of Applied Mathematics and Mechanics, Perm National Research Polytechnic University, 29 Komsomolsky Prospekt, 614990 Perm, Russia

**Keywords:** deep learning, explainable artificial intelligence, predictive maintenance, prognostic and health management

## Abstract

A classic problem in prognostic and health management (PHM) is the prediction of the remaining useful life (RUL). However, until now, there has been no algorithm presented to achieve perfect performance in this challenge. This study implements a less explored approach: binary classification of the state of mechanical systems at a given forecast horizon. To prove the effectiveness of the proposed approach, tests were conducted on the C-MAPSS sample dataset. The obtained results demonstrate the achievement of an almost maximal performance threshold. The explainability of artificial intelligence (XAI) using the SHAP (Shapley Additive Explanations) feature contribution estimation method for classification models trained on data with and without a sliding window technique is also investigated.

## 1. Introduction

Maintenance of mechanical systems requires an approach that allows one to make decisions in order to improve their productivity, efficiency, and safety. In general, there are several approaches to maintenance. The first approach is often referred to as reactive maintenance (RM) [1]. Researchers also use the definition from [2] and define it as corrective maintenance (CM). These definitions are essentially the same—this is an approach in which parts are replaced as they fail. It provides full usability of parts, but requires a mechanical system to suffer downtime, and therefore tends to cause serious delays and high unscheduled repair costs.

Preventive maintenance (PM) is an approach in which it is possible to predetermine the useful life of a part and maintain or replace it before it fails. Preventive maintenance avoids unplanned failures but incurs costs for planned downtime and underutilization of the component.

Predictive maintenance (PdM) is an approach that aims to optimize the balance between RM and PM by replacing components in a timely manner. With this approach, mechanical systems are maintained only when they are close to failure and, consequently, component life is extended (compared to PM) and unplanned maintenance and labor costs are reduced (compared to RM). Ultimately, solving PdM tasks leads to the optimization of periodic maintenance, the minimization of downtime and, as a consequence, the prevention of property damage. The concept of PdM has existed for many years, but only recently emerging technologies have become effective and affordable enough to make PdM widely available [3]. As indicated in [4], a sharp increase in the number of publications that mention PdM began in 2012. It is noteworthy that the number of works related to artificial intelligence also began to grow exponentially in the same decade.

For example, autoencoders have been successfully used for feature extraction [5], multi-sensory data fusion [6], fault diagnosis [7,8], and anomaly detection [9] because they do not require prior knowledge of the data and can compress and fuse multi-sensory data. Autoencoders are not as efficient at reconstruction compared to generative adversarial networks (GANs) [10], which allows GANs to solve the class imbalance problem [11,12]. However, it is difficult to stabilize the training of GANs and teach them to generate discrete data. Due to their ability to detect important features, convolutional neural networks (CNNs) have also found applications in predicting remaining useful life (RUL) [13,14] and fault diagnosis [15,16,17]. However, CNNs can be easily overfit on time series, so recurrent neural networks (RNNs), which are good at capturing consistent dependencies over time, are mostly used in the same tasks of fault diagnosis [18,19,20] and RUL prediction [21,22,23]. Nevertheless, RNNs are not without drawbacks: problems with vanishing and exploding gradients can occur, as well as difficulties in processing very long sequences. The previously presented works on PdM can be roughly divided into two areas: diagnostic and prediction. Diagnostics involves detecting anomalies, finding the root cause of failures and analyzing the current state of the system. Prediction aims at predicting the values of the set point, the RUL and the future state of the system. This paper is devoted to the prognostic direction.

Currently, there are plenty of works devoted to designing neural network models that can improve the quality of RUL prediction: using multilayer perceptron [24], convolutional [24,25] and recurrent neural networks [26,27], as well as hybrid models [28,29]. There is a limitation in the predictive ability of neural networks, which does not allow the prediction error of RUL to be reduced below a certain level. For example, the best achieved result in RUL prediction on the C-MAPSS dataset (subset FD001) [30] is 11.18 using the root-mean-square error (RMSE) metric [31].

However, the RUL prediction problem can be reformulated into a binary classification problem for the state of the system as a whole at a given prediction horizon, and there are several advantages of using such a formulation. Firstly, there are models that may not give state-of-the-art results in RUL prediction, but will achieve the highest quality in the classification problem (which is confirmed by the present work with the achieved 99% accuracy). Consequently, using simpler models to achieve better results has the following advantages: lower computational resource requirements, simplified model implementation, and less time needed to train the neural network, obtain the prediction, and adjust hyperparameters. Secondly, the transition from RUL to probability of failure will expand the possibilities of interpreting the model in terms of a more comprehensible and accessible measure of the system state—a probabilistic measure. This statement is particularly important, because the interpretability of neural network models plays an equally important role, since understanding why a neural network produces certain predictions can be a crucial factor in making decisions about maintenance.

A study on the application of explainable artificial intelligence (XAI) methods from the PdM point of view has shown that the SHAP (Shapley Additive Explanations) additive interpretation method has the best stability and consistency in its results [32]. In addition, SHAP generates explainable trajectories, which have the properties of monotonicity, trendability and predictability [33]. This means that SHAP values can be trusted to explain feature contribution or feature importance. However, the interpretability of neural network models is still considered only in very few publications that include a mention of PdM [34]. Even among them, the issue has been raised only for models of RUL prediction [35,36] and anomaly detection [9]. It was also observed that the analysis of the contribution of features was often limited to the calculation of SHAP values only, without any hypotheses being put forward and confirmed.

Thus, the purpose of this work is to develop a mechanism for making rational decisions on the maintenance of mechanical systems on the basis of intelligent analysis of the time series of indications obtained by the sensors. To achieve this goal, in addition to solving the problem of RUL prediction, we propose investigating, in terms of interpretability and predictive ability, the new approach—binary classification of the state of mechanical systems on a given prediction horizon.

This paper is organized as follows: Section 2 describes the problem formulation. Section 3 contains a description of the neural network architecture and hyperparameters for training. Section 4 describes in detail the dataset used to test the new approach and presents the prediction results. Finally, Section 5 is devoted to a discussion of the interpretability of the classification model.

## 2. Problem Statement

To assess the technical state of mechanical systems, two formulations of the problem are proposed, and the results of both are presented in the current work.

### 2.1. Regression

Prediction of remaining useful life (RUL) represents a regression problem and is based on the following hypotheses:

- The number of cycles remaining to the moment of failure must be obtained from the system sensors;
- The area of definition of RUL is the set $\left\{0\right\}\cup \mathbb{N}$;
- The selected loss function is the mean-square error

(1) $$MSE=\frac{1}{n}\sum_{i=1}^{n}{\left(y_{i}-{\hat{y}}_{i}\right)}^{2},$$

where $y_{i}$ represents the true RUL values, $\hat{y}$ presents the predicted RUL values, and n is the total number of observations.

### 2.2. Binary Classificaton

Binary classification of system failure at a given forecast horizon is performed based on the following hypotheses:

- The system failure will be understood as the moment from which the system becomes unsuitable for further use;
- The forecast horizon is 30 time units;
- It is necessary to know the probabilities of systems belonging to the positive class (will fail);
- Binary classification is performed: “0”—the system will not fail at a given forecast horizon, “1”—the system will fail;
- The boundary between classes is determined by the threshold value μ. If the probability of the system that belong to the positive class (object $x_{i}^{\prime }$ from the test sample $X^{\prime }$) $p_{i}$ is lower than μ, then the system is assigned to class «0», otherwise «1»:

(2) $$x_{i}^{\prime }\to \left\{\begin{array}{cc} 1, & p_{i}\geq \mu \\ 0, & p_{i}<\mu \end{array}\right..$$

- The threshold value is determined as follows:

(3) $$\mu \left(\alpha^{*}\left(X^{\prime }\right)\right)=arg\underset{\mu^{\prime }\in M}{min}G\left(\mu^{\prime },\alpha^{*}\left(X^{\prime }\right)\right),$$

where M is a set of rational numbers from 0 to 1, $\mu^{\prime }$ is some value from M, and G is the quality predictive function of the algorithm $\alpha^{*}$.

- The selected loss function is binary cross-entropy

(4) $$L\left(a,X\right)=\frac{1}{n}\sum_{i=1}^{n}-\left(y_{i}\times log\left(p_{i}\right)+\left(1-y_{i}\right)\times log\left(1-p_{i}\right)\right),$$

where n is the number of observations, $y_{i}$ is a binary indicator (“0” or “1”), $p_{i}$ the predicted probability that the system state belongs to the positive class.

## 3. Solution Method

The problem is solved by training a bidirectional recurrent neural network (BiRNN) with long short-term memory (LSTM) [37], which will be referred to as BiLSTM below.

### 3.1. BiLSTM

All temporal steps of the input sequence (unlike the problems that require output after each input or at the end of some input segment [38]) are available to solve the problems stated in this paper. Consequently, it is possible to use a bidirectional recurrent neural network to take into account the flow of information not only in the positive but also in the negative time direction [39].

**Figure 1:**
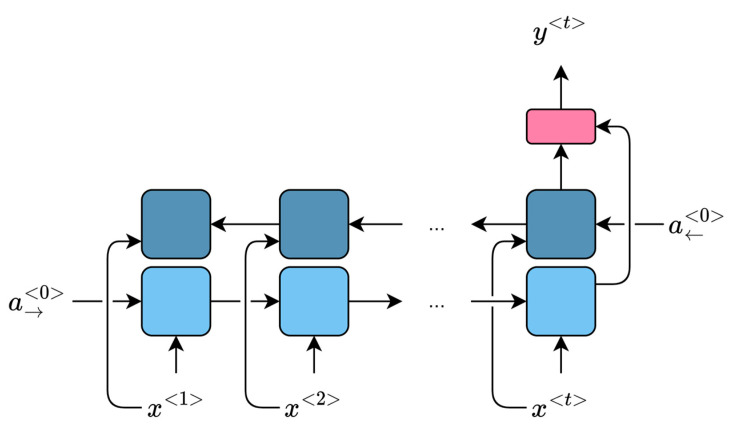
Many-to-one type of single-layer BiRNN unrolled over time. Here, $x^{\langle t\rangle }$ is the input at time step t, $y^{\langle t\rangle }$ is the output at time step t, and $a_{\to }^{\langle 0\rangle }$ and $a_{\leftarrow }^{\langle 0\rangle }$ are activations at the initial time step in the forward and reverse directions of the RNN, respectively.

The task of classification of the system failure at a given prediction horizon, as well as the task of RUL prediction, requires only one value on the output of the neural network. Therefore, in this paper, the many-to-one RNN type is used (Figure 1).

### 3.2. Model and Hyperparameters

The model presented in Table 1 is trained using an Adam optimizer ($\beta_{1}=0.9$, $\beta_{2}=0.999$) [40] with a learning rate of $10^{-5}$ and a batch size of 32. After the first two fully connected layers, a dropout layer [41] is used with a drop fraction of units of 0.2. The output is a fully connected layer with one neuron, which has a linear activation function for the RUL prediction task and a sigmoid for the classification task.

## 4. Results and Discussion

### 4.1. Input Data

As an example, one of the most popular publicly available datasets for solving predictive tasks to assess the technical state of mechanical systems is used to solve both problems. It contains the sample values of features (Table 2) from the damage simulation model of a hundred aircraft engines. The damage simulation and data synthesis were performed by the authors of [30]. The entire dataset is divided into four subsets, but the present study uses a single subset named FD001. The following will describe how the authors of [30] modeled damage and synthesized the data contained in the FD001 subset.

**Figure 2:**
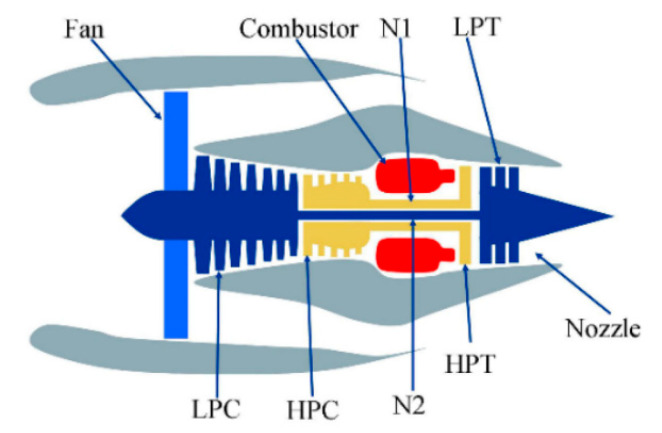
Simplified engine diagram in C-MAPSS [42]. Rotating modules: fan, low-pressure compressor (LPC), high-pressure compressor (HPC), high-pressure turbine (HPT), low-pressure turbine (LPT).

At first, a model was chosen that would allow the input of changes in parameters related to the state of the engines and save the results of sensors measurements. The C-MAPSS software [42], which allows simulation of the operation of a two-circuit turbofan engine, met these requirements. To check the adequacy of the aircraft engine model, response surfaces were built, which investigate the relationship between flow and efficiency losses of individual rotating engine components (Figure 2) in relation to the parameters for health index calculation for these components. The health index will be understood as a quantitative indicator of the engine state at time t, described by Formula (5).

After studying such degradation models as Arrhenius, Coffin–Manson fatigue crack growth, and Eiring, it was noticed that the exponential law for time to failure that is common for all models is $t_{fault}=Ae^{B\left(t\right)}$, where A is the scaling factor and $B\left(t\right)$ is some function depending on the considered degradation process. That and the observation of similar degradation trends in practice motivated them to apply this law for modeling changes in state parameters and calculating the health index:

(5) $$H\left(t\right)=min\left(e\left(t\right),f\left(t\right)\right);$$

(6) $$e\left(t\right)=1-d_{e}-e^{a_{e}\left(t\right)t^{b_{e}\left(t\right)}};$$

(7) $$f\left(t\right)=1-d_{f}-e^{a_{f}\left(t\right)t^{b_{f}\left(t\right)}},$$

where $d_{e}$ and $d_{f}$ are the initial degree of degradation and manufacturing in terms of efficiency and consumption, respectively, $t^{b\left(t\right)}=B\left(t\right)$, and $e^{a\left(t\right)}=\frac{A}{th_{wear}}$, where $th_{wear}$ is an upper wear threshold that denotes an operational limit beyond which the component/subsystem cannot be used.

Thus, data synthesis was conducted according to the following algorithm:

1. Select the initial degree of degradation (within 1%);
2. Introduce an exponential rate of degradation;
3. Stop data synthesis when the health index reaches zero;
4. Impose noise on the obtained data, taking into account the effects of maintenance between flights and differences in operating conditions (weather parameters, aircraft load, pilot operating style, etc.).

The health index should depend on the condition of each of the rotating assemblies, but in the considered data, the failure criterion is the situation when the residual strength became less than 15% of the original HPC strength, i.e., when $e\left(t\right)<0.15$ or $f\left(t\right)<0.15$.

### 4.2. Preprocessing and Testing

Before the direct training of the neural network, the initial data were pre-processed. It was noticed that the features OS3, S1, S5, S6, S10, S16, S18 and S19 have a constant value during the entire time interval (the full list of measured features is presented in Table 2). These features were removed because they do not carry any useful information. In addition, the values measured by the sensors have different ranges and therefore the values for the features have been scaled to the range $\left[0,1\right]$:

(8) $$x_{scaled}=\frac{x-x_{min}}{x_{max}-x_{min}}.$$

Thus, the total volume of the training set is 20,631 samples. The model was trained using TensorFlow machine learning library [43] and Keras interface [44] on NVIDIA RTX A5000 GPU, AMD Ryzen 9 5900X CPU and 64 GB RAM. The presented results were calculated on the last time step of the sensor indications for each engine; hence, the volume of the test set was 100 samples. It is important to note that the test sample has no cycles in which the engine would fail.

### 4.3. Solution of the RUL Prediction Problem

**Figure 3:**
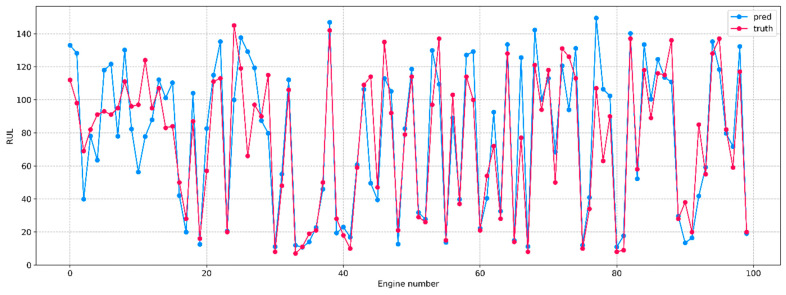
Predicted (blue) and true (red) engine RUL values. Table 3 shows a comparison of the RUL prediction results of the presented model, which will be referred to as BiLSTM (baseline), with neural network architectures from other studies concerning the RMSE metric. The model presented in this paper is similar to the model from [27]. It is assumed that the difference in the results between them is due to the insufficient optimization of hyperparameters. The largest decrease in RMSE occurred after the transition from classical MLP to more modern recurrent and convolutional neural network architectures, while hyperparameter optimization also added a significant gain, but the use of hybrid architectures did not have a great effect. In this regard, there seems to be a performance threshold for RUL prediction on this dataset, so it makes sense to switch from the regression task to the classification task.

By plotting the dependence of predicted and true RUL values on the engine number (Figure 3), it can be found that the model has a high predictive ability. The average absolute error is convenient to use in order to interpret the obtained result:

(9) $$MAE=\frac{1}{n}\sum_{i=1}^{n}\left|Y_{i}-{\hat{Y}}_{i}\right|,$$

where $Y_{i}$ represents the true RUL values, $\hat{Y}$ represents the predicted RUL values, and n is the total number of observations. In this case, the MAE is approximately 14.815, i.e., the model is on average wrong by 15 cycles. It is important to note that most of this error is due to about 10% of the engines whose RUL predictions stand out strongly from the overall picture. Therefore, it can be concluded that for 90 out of 100 engines, satisfactory forecasts of their remaining useful life are obtained.

### 4.4. Solution of the Classification Problem

**Figure 4:**
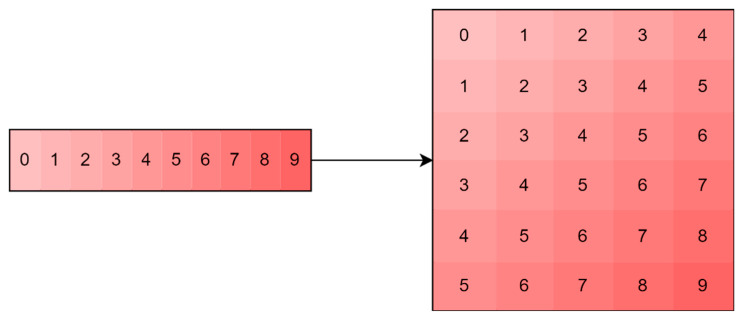
Example of sliding window application (l=5) for a vector of numbers from 0 to 9.

As opposed to the regression model, the training of the classification model was performed not only on the data with the sliding window technique, but also without it. This was done in order to further compare the interpretability of the model trained on simplified and complicated data. By using a sliding window of size l, we observe such a data transformation that splits the original sequence into a set of sequences of length l, which, in turn, is a set of elements included in the sliding window moving with a single step along the original sequence (Figure 4). In this paper, l=21.

**Figure 5:**
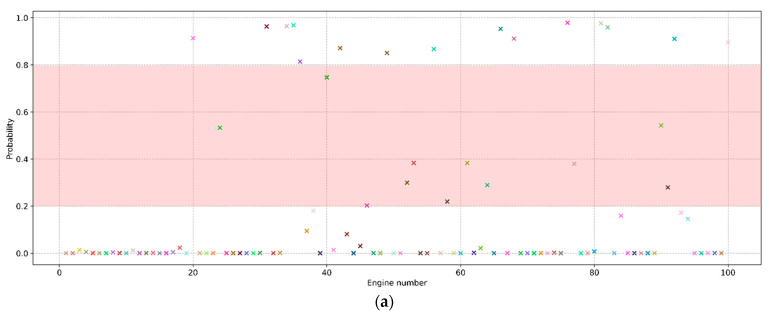

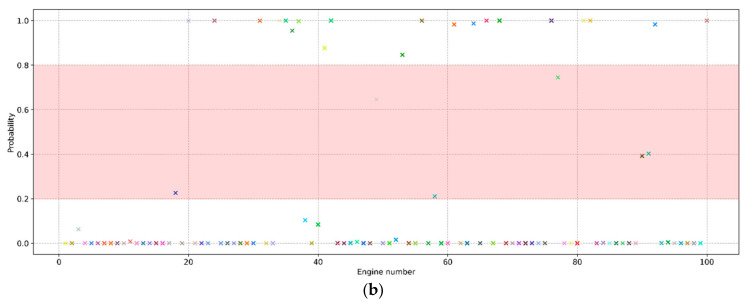
Probabilities of engine failure in the next 30 cycles for the model trained on data without (**a**) and with (**b**) a sliding window. The area highlighted in red contains engines that cannot be assigned to any class with a high degree of certainty.

The obtained probabilities of engine failure from the test sample are shown in Figure 5. It is evident that the training of the neural network on the data using the sliding window technique increases the prediction confidence. However, some engines still cannot be assigned to a particular class with high confidence. To ensure a clear separation into classes, it is necessary to find an optimal threshold value of the probability below which the engine will be assigned to class “0”, and otherwise “1”.

### 4.5. Finding the Optimal Threshold Value

Let us define the basic metrics needed to find the optimal threshold value μ:

- Precision is the fraction of objects called positive by the classifier that are indeed positive:

(10) $$Precision=\frac{TP}{TP+FP},$$

where TP is true positive predictions, FP is false positive predictions (error of the second kind).

- Recall is the fraction of objects of the positive class found by the algorithm out of all objects of the positive class:

(11) $$Recall=\frac{TP}{TP+FN},$$

where FN is false negative predictions (error of the first kind).

- True Positive Rate (TPR) is an analog of Recall.
- False Positive Rate (FPR) is the fraction of negative class objects incorrectly predicted by the algorithm:

(12) $$FPR=\frac{FP}{FP+TN},$$

where TN is true negative predictions.

When working with data on the condition of an aircraft engine, it is reasonable to assume that the correct approach in the selection of *μ* will be to minimize the error of the first kind, because in a real-life situation it is better to double-check the engine than to hope for its correct operation. This will lead to unnecessary financial expenses for diagnostics, but it will reduce the probability of engine failure during operation. With this approach, it is worth giving preference to the fact that the proposed algorithm demonstrates the ability to detect a failure in general, so it is necessary to maximize Recall (or TPR).

The second approach is to find the balance between errors of the first and second kind. For this purpose, an ROC curve can be constructed, which shows the dependence of TPR on FPR with variation of the threshold. Here, the best solution is to choose thresholds that correspond to the minimum of the TPR−FPR difference. However, for samples with class imbalances, this approach usually does not lead to a good result. In addition, in a situation where several minima are found, it is impossible to choose a particular one, since it is unknown whether the total sum of errors of the first and second kind will decrease.

To avoid the disadvantages described above, in order to implement the second approach, instead of minimizing the TPR−FPR difference, let us minimize the F-measure:

(13) $$F_{\beta }=\left(1+\beta^{2}\right)\cdot \frac{precision\cdot recall}{\left(\beta^{2}\cdot precision\right)+recall}.$$

In order to obtain a balance between FP and FN, it is necessary to take a coefficient value β=1:

(14) $$F_{1}=2\cdot \frac{precision\cdot recall}{precision+recall}.$$

Therefore, Precision and Recall will have the same weight in the evaluation of the result.

The results of the two approaches described above are presented in Table 4. It can be seen that for the model trained on sliding window data, the optimal threshold values for the two approaches coincide. In addition, the use of the sliding window technique confirms an improvement in the predictive ability of the neural network, since it becomes possible to select a threshold value at which the model is wrong in only 1 case out of 100. In addition, there is no engine that the model detects as a false negative.

Based on the obtained results of the binary classification of the state of aircraft engines, it can be concluded that the transition from the regression problem to the classification problem turned out to be successful, since it was possible to achieve 99% accuracy. Moreover, a relatively simple model was required to achieve this result, which, firstly, is easier to implement, unlike hybrid models, and, secondly, does not require additional time for hyperparameter optimization.

## 5. Interpretation of the Classification Model

An important step when using neural networks or machine learning algorithms is their interpretation, because understanding the reason why a model makes a certain prediction can be as important as the accuracy of the model. The Deep SHAP method [45] was used to explain the resulting probabilities of aircraft engine failure, which estimates the influence of each of the available features on the model prognosis. The philosophy of this method can be explained by the classical formula for calculating Shapley values:

(15) $$\phi_{i}=\sum_{S\subseteq F\backslash \left\{i\right\}}\frac{\left|S\right|!\left(\left|F\right|-\left|S\right|-1\right)!}{\left|F\right|!}\left[f_{S\cup \left\{i\right\}}\left(x_{S\cup \left\{i\right\}}\right)-f_{S}\left(x_{S}\right)\right],$$

where $\left|F\right|$ is the total number of features from the whole set of features F, S is a subset of features ($\left|S\right|$ is the size of this subset), which does not include the i-th feature, $f_{S\cup \left\{i\right\}}$ represents the model predictions for data $x_{S\cup \left\{i\right\}}$ (with i -th feature), and $f_{S}$ represents the model predictions for $x_{S}$ (without the i-th feature). If the mathematical expectation is taken from the entered predictions, then the value $\phi_{i}$ for the i-th feature will be a constant, and in this case, its positive (negative) value will tell us about the increasing (decreasing) influence of the i-th feature on the probability of failure. The authors [45] also took into account the specificity of neural networks and came up with a way to optimize the calculation of Shepley values by approximating $\phi_{i}$ using Deep LIFT [46].

### 5.1. Enchanced Interpretability

In this section, we consider a model trained on data to which the sliding window technique has not been applied. In this regard, there is an opportunity to conduct a fairly in-depth analysis, which will allow a good understanding of what data the trained model considers important.

**Figure 6:**
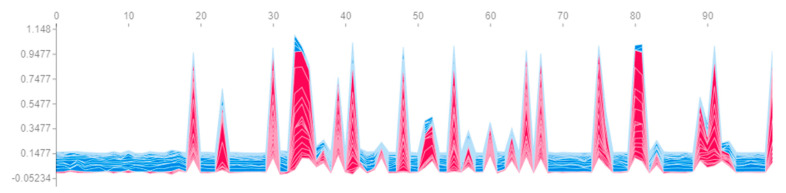
Contribution of all features to the probability of engine failure (red means positive contribution, and blue means negative).

**Figure 7:**
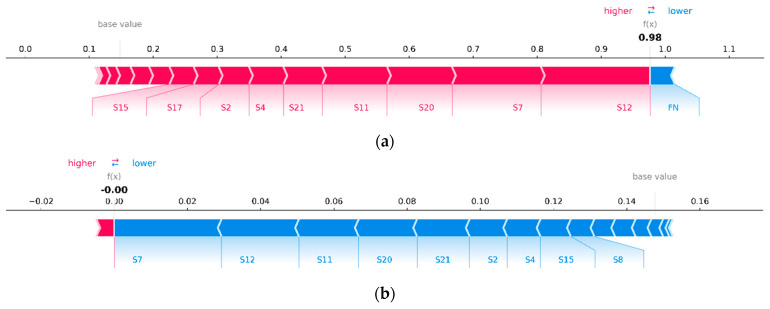
Contribution of features to the probability of failure of engines #81 (**a**) and #99 (**b**), where the base value is the average predicted value on the training set (or the value that would be predicted by the trained model if no features of the test engine were known, i.e., the values of all its features are equal to zero).

First, let us build a graph (Figure 6), the axes and peak values of which exactly correspond to Figure 5, but with the difference being that its interactive version allows us to see the most important features affecting the prediction for a particular engine. The vertical cross-sections of this graph for engines #81 and #99 are presented on Figure 7. The length of each segment, labelled with the name of the feature, denotes its contribution to the probability of engine failure at the output of the neural network $f\left(x\right)$. It is evident that most of the contribution to the probability of failure of engines #81 and #99 consists of the same set of features. Similar patterns can be observed for engines with a probability of failure close to 100% or 0%. Hence, it can be concluded that the same set of features makes the greatest contribution to the model’s confident predictions.

**Figure 8:**
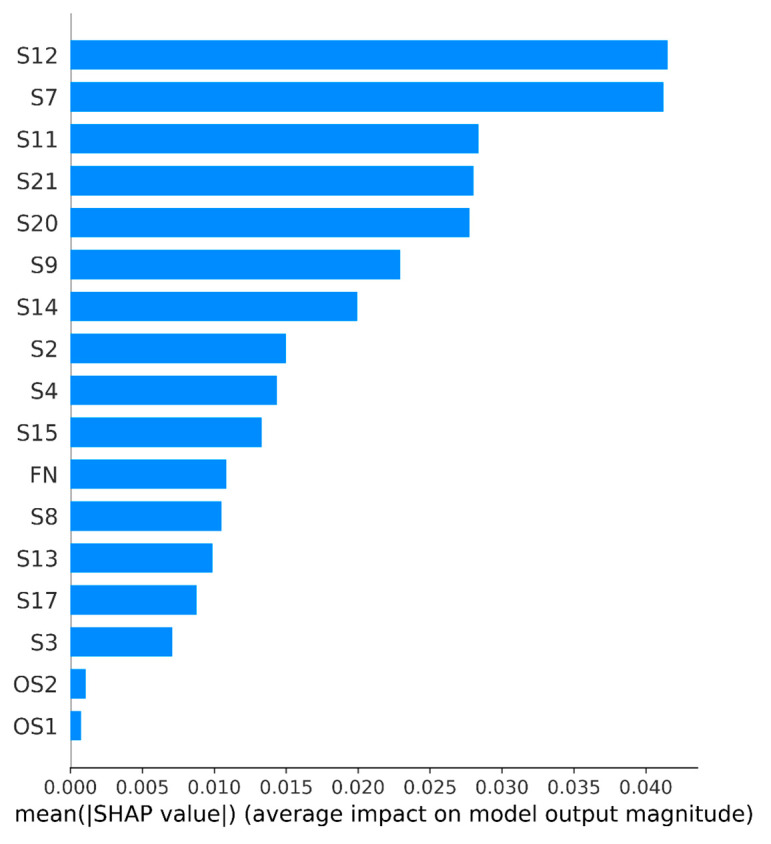
Histogram of the contribution of the features, where the contribution of each feature is taken as the average absolute value of this feature for all engines.

Since not all engines can be confidently assigned to this or that class and they differ from each other in their characteristics and operating parameters, such an analysis can give different results of the contribution of features to the probability of failure. In this case, a ranked list of features can be obtained by taking the modulo average of the contributions (SHAP values) of each feature for all engines (Figure 8). Here, we observe the same set of the most significant features as in Figure 7. This result is explained by the fact that most of the predictions have a probability close to 100% or 0% (see Figure 5). This confirms the previous conclusion about the contribution of the features to confident predictions.

**Figure 9:**
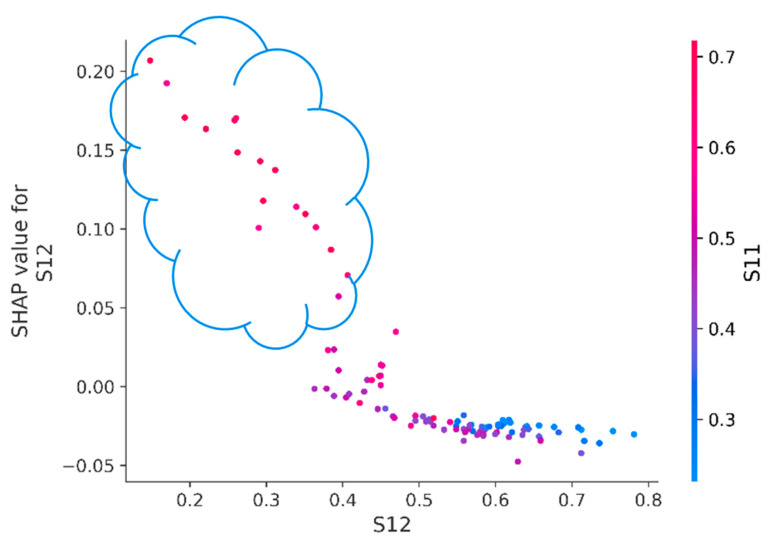
Dependence of SHAP values of the S12 feature on its value (the gradient indicates the value of the S11 feature). A cluster of engines with SHAP values for the S12 attribute higher than 0.025 is highlighted by the cloud.

**Figure 10:**
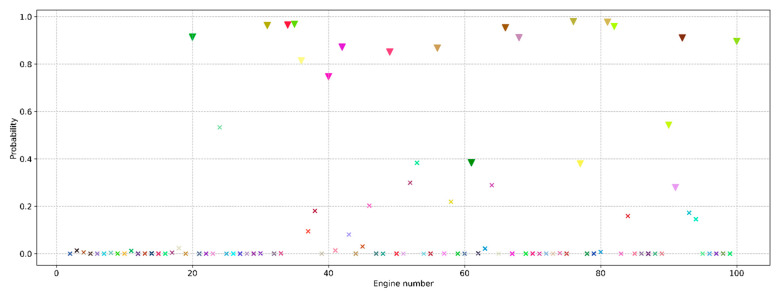
Probabilities of engine failure in the next 30 cycles for the model trained on simplified data, where the triangles are the engines from the considered cluster.

Since it was initially known that S11 and S12 values depend on each other, and in Figure 8 their contribution to the prediction is one of the most significant, it is interesting to look at their dependence directly: in Figure 9, it can be noted that often, high S11 values and low S12 values increase the SHAP value for S12. In other words, this behavior often increases the probability of engine failure. If the cluster of points in the upper left corner are separated by SHAP value above 0.025 and the engines with behavior that describes this dependence are identified, then by marking these engines with triangles in Figure 10, one can see that this behavior mostly refers to engines with a high probability of failure. The only four engines (#61, #77, #91 and #91) stand out strongly from the general rule, with less than 60% probability of failure, so special attention should be paid to them. Referring to the test data labels and finding the engines from the considered cluster, it turns out that 18 out of 20 engines (without #77 and #91) of this cluster would actually fail in the next 30 days; hence, the initially considered dependence (Figure 9) represents important information.

**Figure 11:**
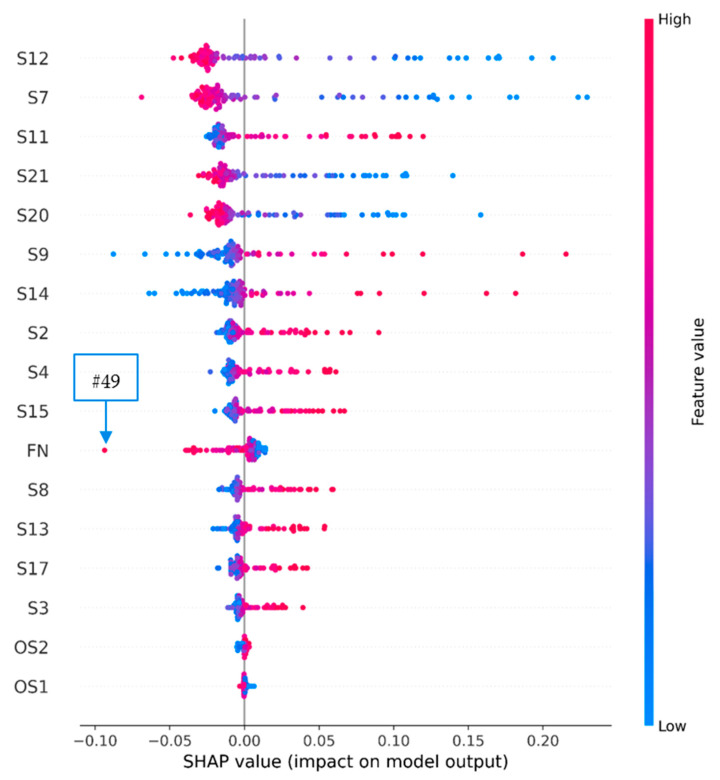
Dependence of the contribution of unique feature values to the forecast on their magnitude (indicated by the blue–red gradient).

Another interesting visualization is shown in Figure 11. Each case of this visualization is represented by a single point for each feature. The position of the point along the abscissa axis is determined by its SHAP value, and the color is used to show the value of the feature value in the point. Here, it is possible to trace the effect of high or low values of a particular feature on the SHAP value. For example, the left tail of the string with SHAP values of the feature FN shows that the neural network does not always consider the long operation of the engine as a sign that in the next 30 days it can fail. The leftmost value of this tail corresponds to engine #49, which the model determined to have an 85% probability of failure. According to the Shepley value, the current cycle number reading of engine #49 reduces its probability of failure by about 10%. This stands out strongly against the general picture for the FN feature (Figure 8) and is due to the fact that the indications of engine #49 were measured most—303 times. Additionally, since no other values of the features for this engine contradict the general pattern, perhaps it should therefore be considered that the probability of failure of engine #49 is in fact higher and it is necessary to pay special attention to it.

Figure 11 also shows that the overall pattern of feature contribution is as follows: high feature values increase the probability of failure while low values decrease it, or low values increase the probability of failure while high values decrease it (OS1 and OS2 features do not fit this general rule). We can also observe points where the value of a feature contributes too much to the prediction. These values often correspond to the engines about which the neural network is unsure regarding their failure, and therefore they need to be considered in more detail.

Thus, thanks to the large number of interpretation tools, knowing the specifics of aircraft engines and analyzing various dependencies, it is possible to achieve high success in identifying inoperable engines. It is important to note that the interpretation tools presented above will not be of any use for models whose quality is too low, since these judgments will refer to the model predictions, and hence there is a high probability of facing the problem of overlapping errors.

### 5.2. Reduced Interpretability

This section examines the interpretation capabilities of a model trained on data to which sliding window technology has been applied. Although the quality metrics and confidence in the technical condition of the engines are higher with this model, the application of the sliding window to the data resulted in a time window dependency being considered when constructing visualization tools, as the number of meaningful (which is not the same size as one) measurements in the data increased.

**Figure 12:**
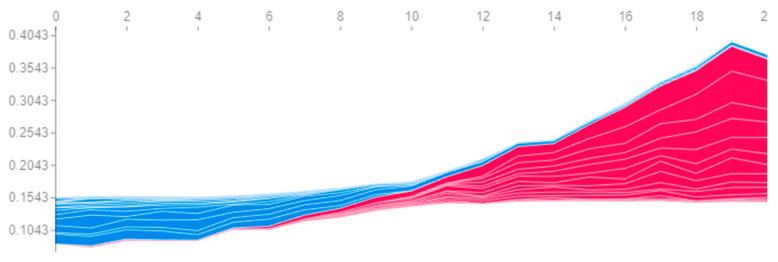
Contribution of features to the probability of failure of engine #81 on time windows.

**Figure 13:**
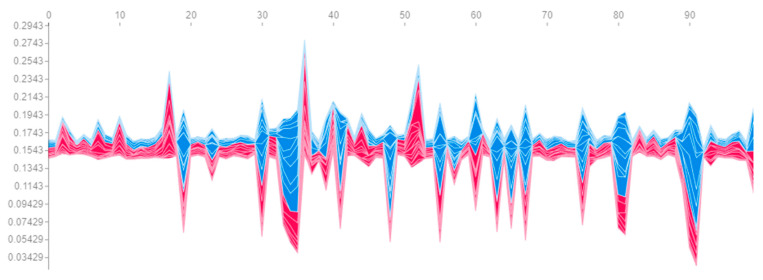
Contribution of features to the probability of engine failure in the last (21st) time window.

In this regard, for example, Figure 6 can be constructed both for a single engine (Figure 12) and simultaneously for all engines within a particular time window (Figure 13). A similar situation occurs with the rest of the visualization tools: complexity arises, which obliges the researcher to take into account the separation by time windows in the data.

**Figure 14:**
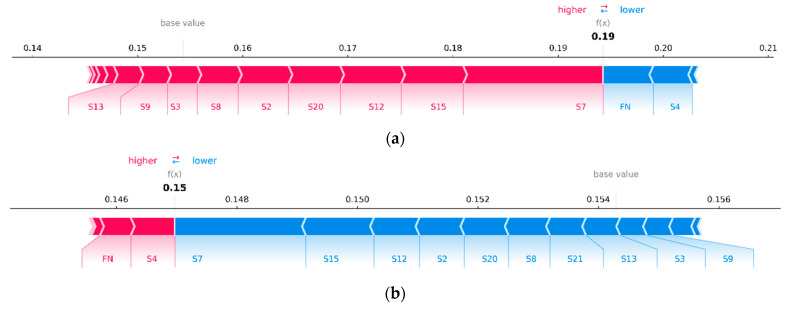
Contributions of features to the probability of engine failure, averaged over all time windows, for the failure of engines #81 (**a**) and #99 (**b**).

To overcome this difficulty, the average SHAP value over all time windows can be calculated. Thus, in Figure 14, we observe a similar picture as in Figure 7: for engines with a high probability of failure, the same features contribute the most, as seen for engines with a low probability of failure, but with the opposite sign. However, in this case, the probabilistic interpretation is lost: $f\left(x\right)$ is no longer equal to the predicted probability of failure of the analyzed engine; hence, it cannot be decomposed into the sum of SHAP values of the features. It should be taken into account that a large contribution of a feature in a particular time window can strongly bias the average estimate if the contribution on the other time windows is small.

**Figure 15:**
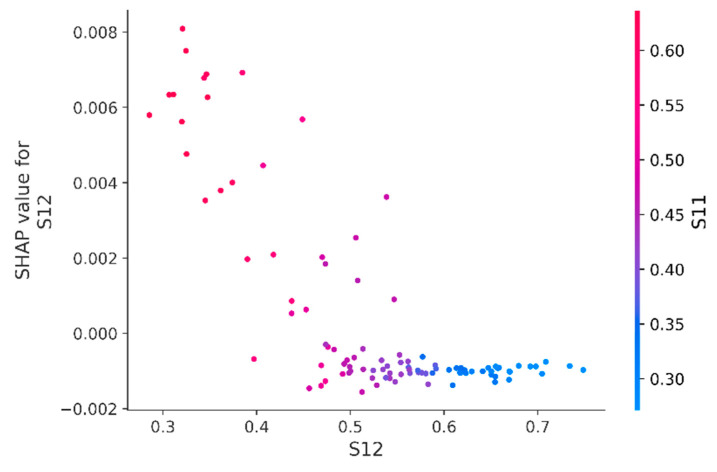
The dependence of the SHAP values of the S12 feature averaged over time windows on its value (the gradient indicates the value of the S11 feature).

Let us consider Figure 15, for which it is more difficult to divide the points into two clusters than in Figure 9. This is because the dispersion of the points has increased and it is impossible to uniquely determine the threshold above which the cluster will be clearly separable, since the area of the described cluster in Figure 9 included points that have a high value of the feature S12, whereas previously the following rule worked: high values of S11 and low values of S12 increase the SHAP value for S12.

Thus, it is still possible to analyze the influence of features on the predictions obtained by the neural network, but only with careful formulation of hypotheses, because the data have a non-trivial dependence on the shift along the time window. In addition, as the size of the time window increases, the complexity of interpreting the visualizations also grows. For example, to obtain the same general representation that was obtained by judging with respect to Figure 11, one would ideally need to plot as many of the same graphs as the sliding window size is initially given, then analyze each of them, consider the relationships between them, and draw a general conclusion. Of course, as shown earlier, the method of averaging SHAP values over all time windows can be applied, but then some of the dispersion in data will be lost (the larger the window size is, the larger amount of dispersion that will be lost). However, the most important drawback is that the probability of engine failure cannot be decomposed into the sum of SHAP values of its features (as it was shown in Section 5.1), due to which the probabilistic interpretation of feature contributions is lost.

## 6. Conclusions

In this paper, the problem of the evaluation of the technical condition of mechanical systems is solved using two methods. The first is one of the most popular to date—predicting the remaining useful life. The results showed that the trained model is wrong on average for all engines by 15 flight cycles. This result is not the best among known works using the C-MAPSS dataset. However, training the same BiLSTM model to solve the problem of binary classification of engine failure at a given forecast horizon resulted in 99% accuracy on the test sample. This suggests that, if necessary, it is possible to reformulate the RUL prediction problem into a classification problem and obtain an efficient, more informative, and less risky model. The possibilities of interpreting classification models by considering the most important features affecting the prediction are also investigated. In addition, the advantages, disadvantages and limitations of the explicable artificial intelligence method SHAP are presented in terms of two cases: when transparency of interpretation is required and when maximum model performance is required.

Thus, on the basis of intelligent analysis of time series of indications of measuring devices, a mechanism has been developed to provide assistance in making rational decisions on maintenance of mechanical systems, and its capabilities have been shown in practice.

## Figures and Tables

**Table 1:** Details of the model used for training.

Layer	Units	Activation
BiLSTM	64	Tanh
BiLSTM	32	Tanh
FC+Dropout (0.2)	16	ReLU
FC+Dropout (0.2)	8	ReLU
FC	1	Linear/Sigmoid

**Table 2:** Transcriptions of the features in the original data.

Feature	Transcription
EN	Engine number
FN	Flight cycle number
OS1	Operation setting #1
OS2	Operation setting #2
OS3	Operation setting #3
S1	Total temperature at fan inlet
S2	Total temperature at LPC outlet
S3	Total temperature at HPC outlet
S4	Total temperature at LPT outlet
S5	Pressure at fan inlet
S6	Total pressure in bypass-duct
S7	Total pressure at HPC outlet
S8	Physical fan speed
S9	Physical core speed
S10	Engine pressure ratio
S11	Static pressure at HPC outlet
S12	Ratio of fuel flow to S11
S13	Corrected fan speed
S14	Corrected core speed
S15	Bypass ratio
S16	Burner fuel-air ratio
S17	Bleed enthalpy
S18	Demanded fan speed
S19	Demanded corrected fan speed
S20	HPT coolant bleed
S21	LPT coolant bleed

**Table 3:** Comparison of method performance on a dataset C-MAPSS (FD001).

Method	RMSE
MLP [24]	37.56
BiLSTM (baseline)	19.12
CNN [24]	18.45
DLSTM [26]	16.14
BiLSTM [27]	13.65
DCNN [25]	13.32
HDNN [29]	13.02
RBM+LSTM [28]	12.56
LSTM-Fusion [31]	11.18

**Table 4:** The result of the two approaches for determining the threshold value for model predictions trained on data with and without a sliding window.

	Precision	Recall	$F_{1}$	FN	FP	FN+FP
$\max\left(TPR\right)$,	0.6944	1	0.8197	0%	11%	11%
μ=0.0136						
(without sliding window)						
$\max\left(TPR\right)$,	0.9615	1	0.9804	0%	1%	1%
μ=0.1034						
(with sliding window)						
$\max\left(F_{1}\right)$,	0.9565	0.88	0.9167	3%	1%	4%
μ=0.289						
(without sliding window)						
$\max(F_{1})$,	0.9615	1	0.9804	0%	1%	1%
μ=0.1034						
(with sliding window)						

## Data Availability

The data presented in this study are available on request from the corresponding author.
